# CT-based nomogram for early identification of T790M resistance in metastatic non-small cell lung cancer before first-line epidermal growth factor receptor-tyrosine kinase inhibitors therapy

**DOI:** 10.1186/s41747-023-00380-7

**Published:** 2023-11-02

**Authors:** Ye Li, Xinna Lv, Yichuan Wang, Zexuan Xu, Yan Lv, Dailun Hou

**Affiliations:** 1 Department of Radiology, Beijing Chest Hospital, Capital Medical University, Beijing, 101149 China; 2 Department of Radiology, Beijing Tuberculosis and Thoracic Tumor Research Institute, Beijing, 101149 China

**Keywords:** Carcinoma (non-small cell lung), ErbB receptors, Nomograms, Radiomics, Tomography (x-ray computed)

## Abstract

**Background:**

To evaluate the value of computed tomography (CT) radiomics in predicting the risk of developing epidermal growth factor receptor (EGFR) T790M resistance mutation for metastatic non-small lung cancer (NSCLC) patients before first-line EGFR-tyrosine kinase inhibitors (EGFR-TKIs) therapy.

**Methods:**

A total of 162 metastatic NSCLC patients were recruited and split into training and testing cohort. Radiomics features were extracted from tumor lesions on nonenhanced CT (NECT) and contrast-enhanced CT (CECT). Radiomics score (rad-score) of two CT scans was calculated respectively. A nomogram combining two CT scans was developed to evaluate T790M resistance within up to 14 months. Patients were followed up to calculate the time of T790M occurrence. Models were evaluated by area under the curve at receiver operating characteristic analysis (ROC-AUC), calibration curve, and decision curve analysis (DCA). The association of the nomogram with the time of T790M occurrence was evaluated by Kaplan–Meier survival analysis.

**Results:**

The nomogram constructed with the rad-score of NECT and CECT for predicting T790M resistance within 14 months achieved the highest ROC-AUCs of 0.828 and 0.853 in training and testing cohorts, respectively. The DCA showed that the nomogram was clinically useful. The Kaplan–Meier analysis showed that the occurrence time of T790M difference between the high- and low-risk groups distinguished by the rad-score was significant (*p* < 0.001).

**Conclusions:**

The CT-based radiomics signature may provide prognostic information and improve pretreatment risk stratification in EGFR NSCLC patients before EGFR-TKIs therapy. The multimodal radiomics nomogram further improved the capability.

**Relevance statement:**

Radiomics based on NECT and CECT images can effectively identify and stratify the risk of T790M resistance before the first-line TKIs treatment in metastatic non-small cell lung cancer patients.

**Key points:**

• Early identification of the risk of T790M resistance before TKIs treatment is clinically relevant.

• Multimodel radiomics nomogram holds potential to be a diagnostic tool.

• It provided an imaging surrogate for identifying the pretreatment risk of T790M.

**Graphical Abstract:**

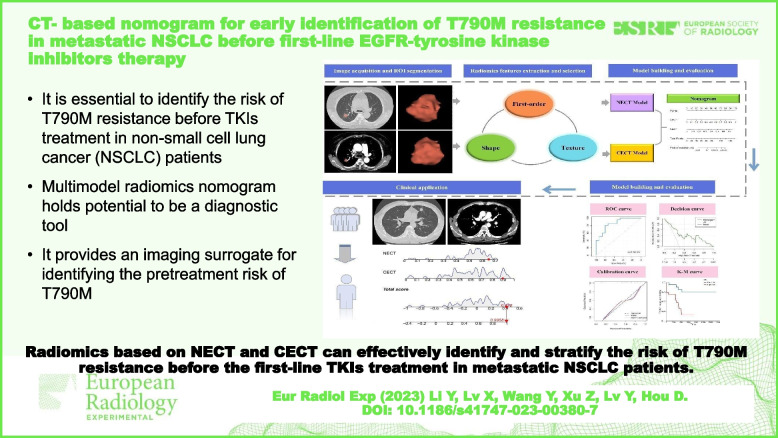

**Supplementary Information:**

The online version contains supplementary material available at 10.1186/s41747-023-00380-7.

## Background

Lung cancer (LC) remains the major cause of cancer-associated mortality in humans, and the cases are rising worldwide [1]. Non-small cell lung cancer (NSCLC) accounting for about 85% of all lung cancers is usually diagnosed at the metastatic stage [2]. For metastatic NSCLC patients, targeted therapy is a main treatment mode [3]. It can reduce tumor burden and prolong the progression-free survival dramatically, especially for those harboring specific somatic genomic alterations [3]. Epidermal growth factor receptor (EGFR) is the most frequent mutation type in NSCLC [1]. In the National Comprehensive Cancer Network guidelines, many first- or second-generation EGFR-tyrosine kinase inhibitors (EGFR-TKIs) are recommended for first-line therapy such as afatinib, gefitinib, and erlotinib [3]. However, unfortunately, it will inevitably develop drug resistance after about 9 to 14 months [3, 4]. T790M is the main cause of acquired resistance, and 60% patients will develop T790M after initial response to first-line EGFR-TKIs [3–6]. Osimertinib has achieved a satisfactory effect on T790M-positive patients when they progressed on first- or second-generation TKIs [5, 7]. Therefore, in order to avoid disease progression, T790M resistance should be identified timely and accurately. But in practical clinical applications, the time of developing resistance ranges from different NSCLC patients. It is of great benefit to formulate an individual treatment strategy if the risk of T790M can be predicted at the same time as metastatic NSCLC being detected.

Reports have suggested that plasma genotyping known as liquid biopsy can be considered to detect and monitor T790M, but the concentration of circulating tumor DNA in blood is relatively low which may lead to false-negative results [8]. Invasive tissue biopsy is needed when plasma testing is negative [9], whereas both liquid and tissue biopsies only reflect the genetic mutation status at the time and do not have predictability.

Radiomics is a method that enables noninvasively evaluating the whole lesion as well as reflecting the microenvironment of the tumor and thus predicting prognosis [10, 11]. Previous studies have revealed that machine learning models constructed by features extracted from metastatic lesions using multisequence MRI can differentiate NSCLC patients with T790M resistance [12, 13]. However, there is a lack of research in exploring the ability of computed tomography (CT) radiomics to stratify metastatic NSCLC patients according to the risk of T790M emergence.

This study devoted to construct and validate a nomogram relied on nonenhanced CT (NECT) and contrast-enhanced CT (CECT) images to predict T790M emergence within 14 months in metastatic NSCLC patients who are diagnosed at the initial time and received the first-line TKIs. And then we extended the follow-up time, to evaluate the stratification ability of the radiomics nomogram.

## Methods

### Participants

This current study was approved by the institutional review board of local hospital. The institutional review board authorized all data in this research for retrospective analysis and waived the demand of informed consent.

The patient’s inclusion criteria were as follows: (1) initial diagnosis with metastatic NSCLC, (2) confirmation of EGFR mutation, (3) treatment with the first- or second-generation TKIs after diagnosis, (4) received T790M testing within 14 months, and (5) availability of baseline CT scans within 1 month before treatment. The exclusion criteria were as follows: (1) poor quality of CT images (presence of artifacts), (2) unclear margin of lesion, (3) incomplete clinical data, and (4) previously received other treatment(s).

**Fig. 1:**
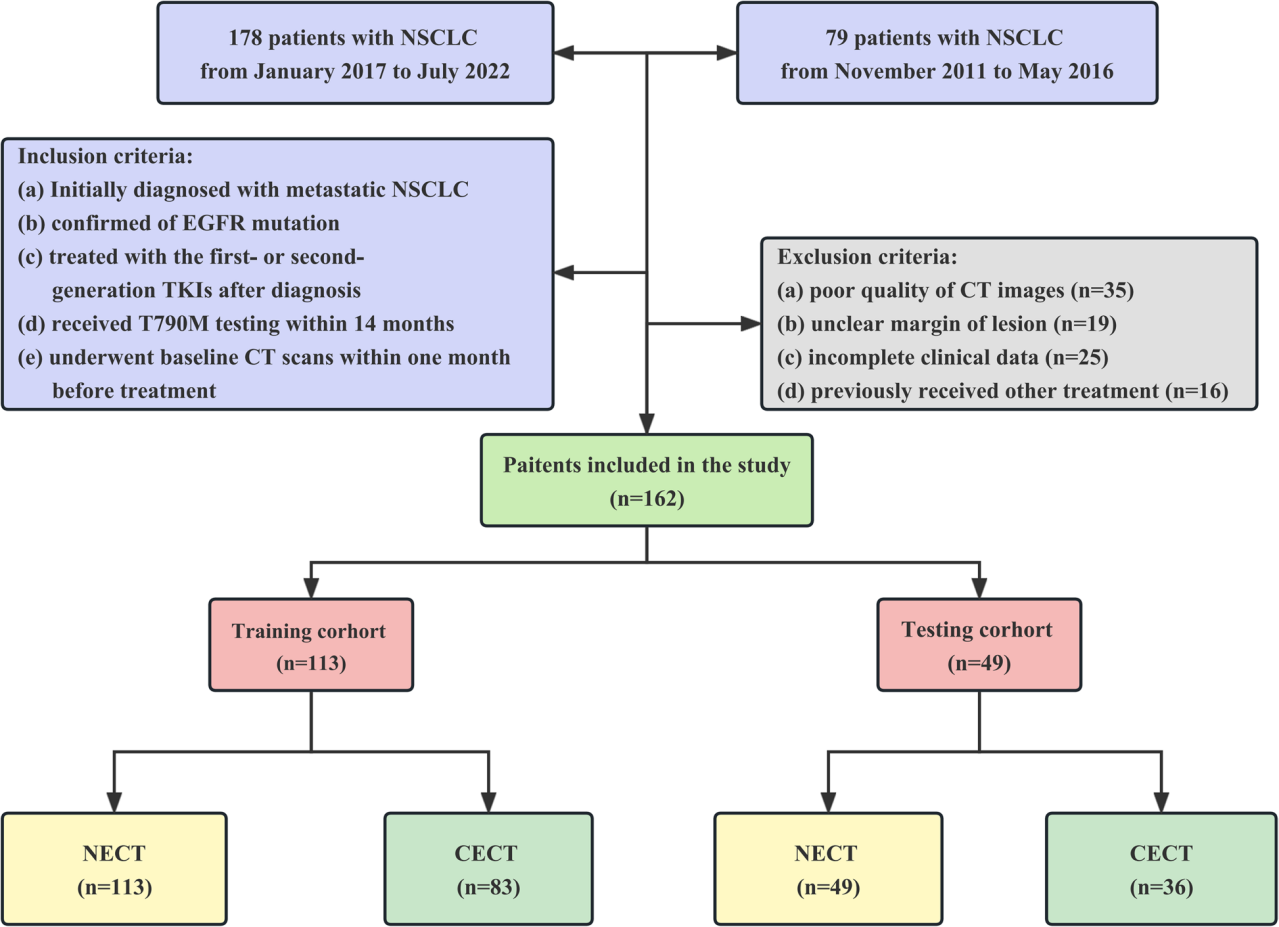
Flowchart of patient selection. For abbreviations, see the Abbreviations list

Finally, we recruited 113 metastatic NSCLC patients from January 2017 to July 2022 as the training cohort and 49 patients from November 2011 to May 2016 as the testing cohort. Totally, there were 162 metastatic NSCLC patients in this research with all patients had NECT images, and 119 of them (73.5%) had CECT images, including 83 patients who emerged T790M within 14 months after TKIs and 79 patients who did not develop T790M resistance in the meanwhile. Figure 1 shows the patient’s enrollment.

### CT protocol

Pretreatment CT images were acquired with one of the following scanners: LightSpeed VCT, Revolution CT, or Optima CT680 system (General Electric Healthcare, Waukesha, WI, USA). The scanning included imaging from the apex pulmonis to basis pulmonis with deep inspiration breath-hold. Scanning parameters were as follows: tube voltage 120 kV, automatic tube current modulation, rotation time 500 ms, pitch 1.375, matrix 512 × 512, window width/window level 1,500/ -500 HU (lung window), and 400/40 HU (mediastinal window). The image reconstruction parameters of slice thickness were 1.25 to 1.5 mm. The CECT scan was performed after NECT and intravenous injection of contrast agent (1.5 mL/kg, flow rate 2.5–3 mL/s). The arterial phase of CECT was acquired 28 s after injecting a contrast agent.

### Image segmentation

Two radiologists with at least 5-year experience in chest CT cooperated in manually drawing the region of interest (ROI) of lesions on the two CT scans (NECT and CECT) layer by layer using 3D slicer (http://www.slicer.org). The ROI was drawn by one experienced chest radiologist and confirmed by the other one. The definition of the ROI was relied on the lung (1,500/-500 HU) and mediastinal window (400/40 HU). They were blinded to the final results of T790M emergence.

### Image preprocessing and radiomics feature extraction

To improve the reproducibility of radiomics features and reduce differences such as various CT scanners, we firstly performed CT image preprocessing by four steps: (a) to ensure the correct calculation of radiomics features, we resampled all CT voxels to 1 × 1 × 1 mm^3^ in-plane resolution; (b) grayscale discretization was applied to normalize intensity of the image signal by a fixed bin width of 25 HU; (c) wavelet (low bandpass filter and high bandpass filter in the *x*, *y*, and *z* directions) and Laplacian of Gaussian filter (sigma 1.0 to 5.0) transformed images were performed to eliminate interference signals; and (d) the z-score transformation as the package default image normalization was applied to standardize the radiomics features to mitigate multimachine effect.

The radiomics features were extracted from ROI based on original and filtered images using the PyRadiomics package of Python. The radiomics features mainly included first-order features, shape and size features, and texture features. Detailed information of the radiomics features can be found in the PyRadiomics official documentation (https://pyradiomics.readthedocs.io/en/latest/features.html).

### Process of radiomics feature selection

Firstly, 35 patients (20 patients without T790M emergence and 15 patients with T790M emergence) were randomly selected and resegmented by another radiologist with 20 years of experience in lung CT for evaluating intraobserver reliability. Features based on the segmentations of two radiologists were compared to evaluate the inter-observer reproducibility. The inter-observer intraclass correlation coefficients > 0.80 of radiomics features were retained. Then the MinMaxScaler method was used to normalized radiomics features in the training and testing cohort. After that, maximal information coefficient was applied to select features considering its capacity of capturing extensive relationships, both functional and not and both linear and nonlinear, in large-scale omics data. We selected the top 200 features based on the maximal information coefficient values which were ranged in decreasing order. Finally, the least absolute shrinkage and selection operator algorithm was performed to select the optimal features with tenfold cross-validation. Radiomics scores (rad-score) of NECT and CECT were calculated by the selected features and their corresponding coefficients which were obtained by least absolute shrinkage and selection operator algorithm. In addition, the detailed formula was listed in Supplementary Material. These steps of feature selection were performed with the Python scikit-learn package (version 3.8, scikit-learn Version 0. 21, http://scikit-learn.org/).

### Radiomics model construction

**Fig. 2:**
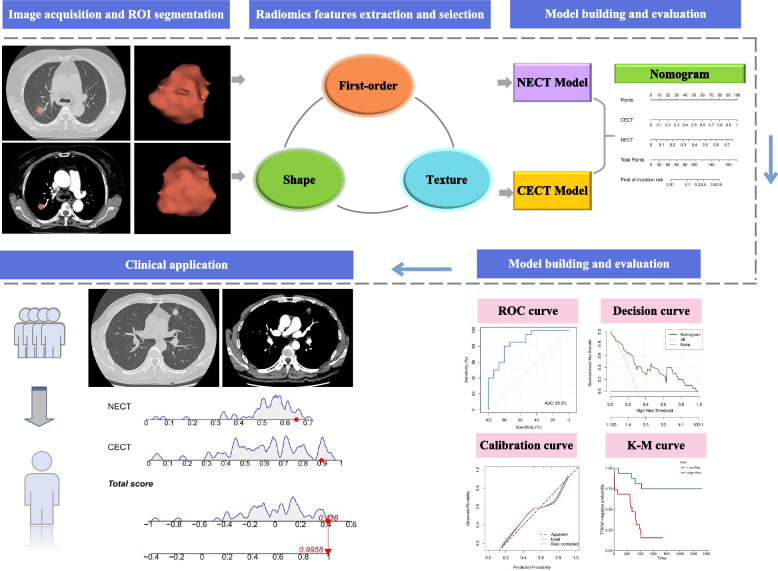
The workflow of this study. For abbreviations, see the Abbreviations list

The selected radiomics features extracted from NECT and CECT images were used to build NECT and CECT models, respectively. These two models were developed and validated using the logistic regression classifier in the training cohort. The rad-score of each patient was evaluated by the combination of selected radiomics features and their relevant weights. Then we constructed a nomogram by incorporating the rad-score calculated by NECT and CECT models. All models were trained and validated in the training cohort which were randomly divided into two parts by the ratio of 7:3 using tenfold cross-validation. By selecting the best model on cross-validation of each model, we tested it in the testing cohort, separately. The process of model construction was applied by the Python scikit-learn package and R software (version 4.2.2). The whole radiomics workflow pipeline was displayed in Fig. 2.

### Statistical analysis

Statistical analysis was performed with SPSS software (version 26) and R software. The independent two-sample *t*-test was used to analyze continuous variables, whereas differences in categorical variables were analyzed using *χ*^2^ test. The discriminative efficacy of these three models was assessed through area under the curve at receiver operating characteristic analysis (ROC-AUC). The specificity, sensitivity, positive predictive value (PPV), and negative–positive value (NPV) were also calculated, respectively. A calibration curve was used to evaluate the consistency between the predicted and actual probability of occurrence of the T790M positive. The decision curve analysis (DCA) was conducted to estimate the net benefits of three models for a range of threshold probabilities. Patients in the independent testing cohort were divided into high- and low-risk groups based on the optimal threshold value which was determined by the Youden index in the training cohort. The association of the risk estimated by nomogram and the time of T790M occurrence was performed to evaluate the prognostic potential of the nomogram. The potential association was evaluated by the Kaplan–Meier survival analysis, the log-rank test, and C-index. The statistical significance levels were all set at *p* < 0.05.

## Results

### Patient baseline clinical characteristics

We collected and analyzed the value of baseline clinicopathologic characteristics for predicting the risk of emerging T790M resistance. There were no significant differences between the two groups in terms of many factors as shown in Table 1, except for the initial EGFR mutation types. However, it only has statistical significance in the total cohort, but not in the training and testing cohort (training cohort, *p* = 0.254; testing cohort, *p* = 0.062).

**Table 1:** Patients and their baseline clinical characteristics before the first-line treatment with EGFR-TKIs

Characteristic	Total cohort (*n* = 162)	T790M positive (*n* = 83)	T790M negative (*n* = 79)	*p*-value
Chest computed tomography, *n* (%)				
Nonenhanced	162 (100.0)	83 (100.0)	79 (100.0)	-
Contrast enhanced	119 (73.5)	67 (80.7)	52 (65.8)	
Histologic type, *n* (%)				
Adenocarcinoma	158 (97.5)	82 (98.8)	76 (96.2)	0.288
Others	4 (2.5)	1 (1.2)	3 (3.8)	
Age years (mean ± SD)	57.8 ± 9.4	57.5 ± 9.3	58.1 ± 9.5	0.685
Gender, *n* (%)				
Male	53 (32.7)	26 (31.3)	27 (34.2)	0.699
Female	109 (67.3)	57 (68.7)	52 (65.8)	
Smoking, *n* (%)				
Yes	41 (25.3)	19 (22.9)	22 (27.8)	0.468
No	121 (74.7)	64 (77.1)	57 (72.2)	
Alcohol consumption, *n* (%)				
Yes	28 (17.3)	14 (16.9)	14 (17.7)	0.886
No	134 (82.7)	69 (83.1)	65 (82.3)	
Initial EGFR mutation, *n* (%)				
L858R	60 (37.0)	35 (42.2)	25 (31.6)	0.018
19Del	92 (56.8)	47 (56.6)	45 (57.0)	
Others	10 (6.2)	1 (1.2)	9 (11.4)	
First-line EGFR-TKI, *n* (%)				
Gefitinib	40 (24.7)	20 (24.1)	20 (25.3)	0.401
Icotinib	102 (63.0)	50 (60.2)	52 (65.8)	
Afatinib	8 (4.9)	4 (4.8)	4 (5.1)	
Erbtinib	12 (7.4)	9 (10.9)	3 (3.8)	
T stage, *n* (%)				
T1	37 (22.8)	18 (21.7)	19 (24.1)	0.118
T2	45 (27.8)	17 (20.5)	28 (35.4)	
T3	16 (9.9)	10 (12.0)	6 (7.6)	
T4	64 (39.5)	38 (45.8)	26 (32.9)	
N stage, *n* (%)				
N0	36 (22.2)	16 (19.3)	20 (25.3)	0.074
N1	7 (4.3)	1 (1.2)	6 (7.6)	
N2	71 (43.8)	36 (43.4)	35 (44.3)	
N3	48 (29.7)	30 (36.1)	18 (22.8)	
M stage, *n* (%)				
M1a	35 (21.6)	19 (22.9)	16 (20.3)	0.147
M1b	46 (28.4)	18 (21.7)	28 (35.4)	
M1c	81 (50.0)	46 (55.4)	35 (44.3)	
Stage, *n* (%)				
IVa	81 (50.0)	37 (44.6)	44 (55.7)	0.157
IVb	81 (50.0)	46 (55.4)	35 (44.3)	

Differences were assessed by independent *t*-test or *χ*^2^ test. *EGFR-TKI* Epidermal growth factor receptor-tyrosine kinase inhibitor, *SD* Standard deviation

### Selection of radiomics features

A total of 4 and 10 radiomics features were obtained from the NECT and CECT after the process of feature selection, respectively.

**Fig. 3:**
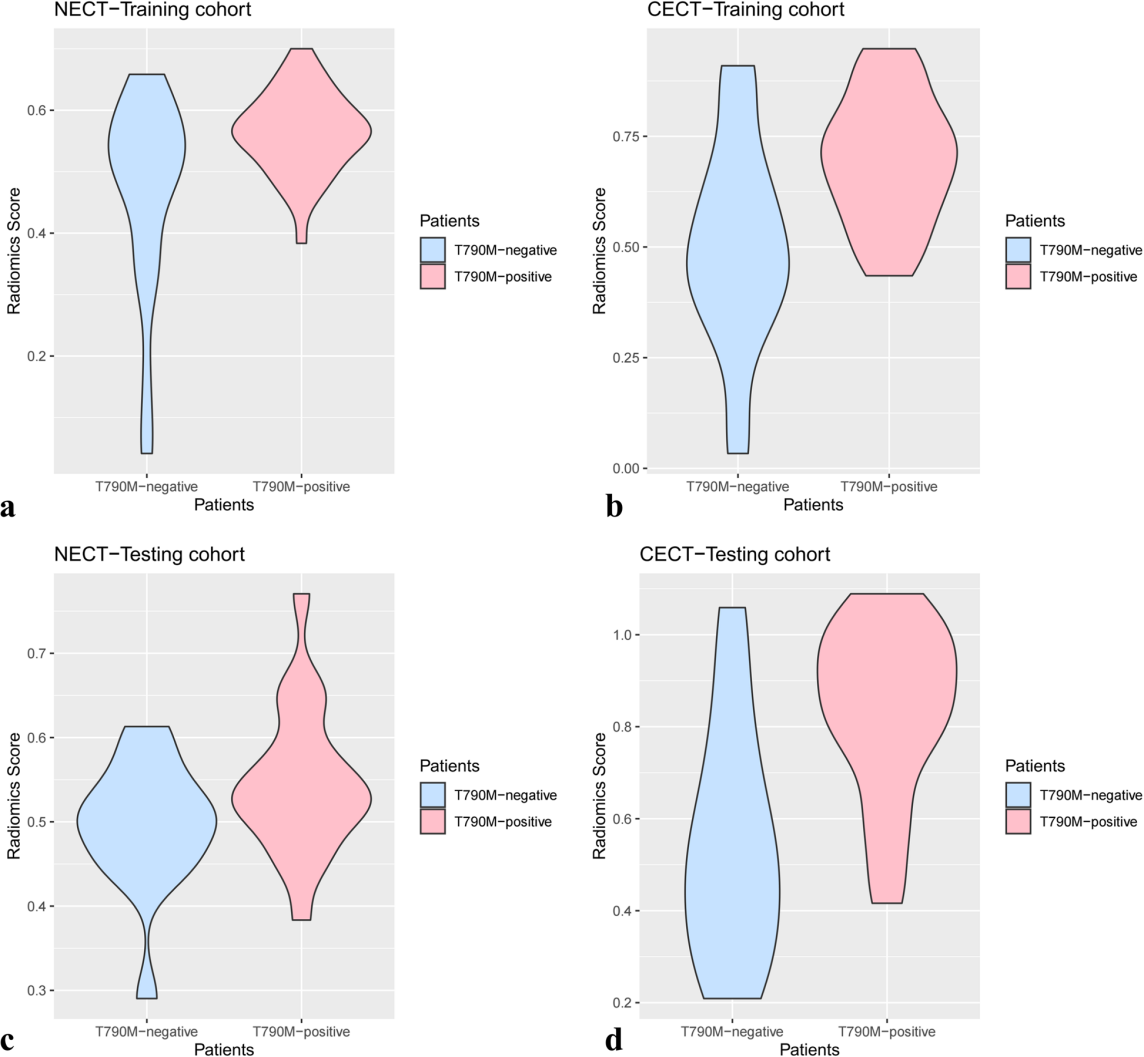
The rad-score of two groups in the training and testing cohort of two CT scans

The rad-score of NECT were significantly higher in the T790M-positive than the T790M-negative group in the training cohort (*p* = 0.013). However, there was no significant difference in testing cohort (*p* = 0.088). In addition, for the rad-score of CECT, the T790M-positive group was significantly higher than the T790M-negative group whether in the training (*p* < 0.001) or testing (*p* = 0.001) cohort (Fig. 3).

### Model performance

**Fig. 4:**
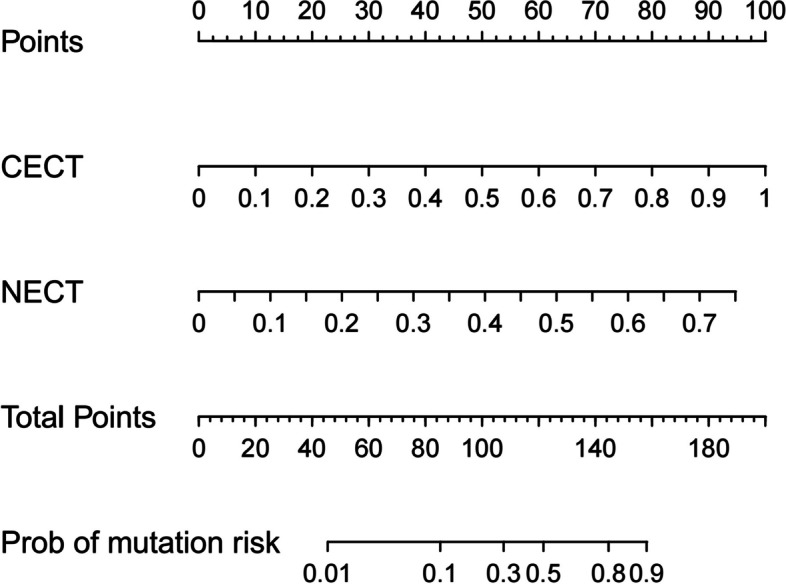
The nomogram incorporating the rad-score of NECT and CECT was developed in the training cohort. *CECT* Contrast-enhanced computed tomography, *NECT* Nonenhanced computed tomography, *Prob* Probability

**Fig. 5:**
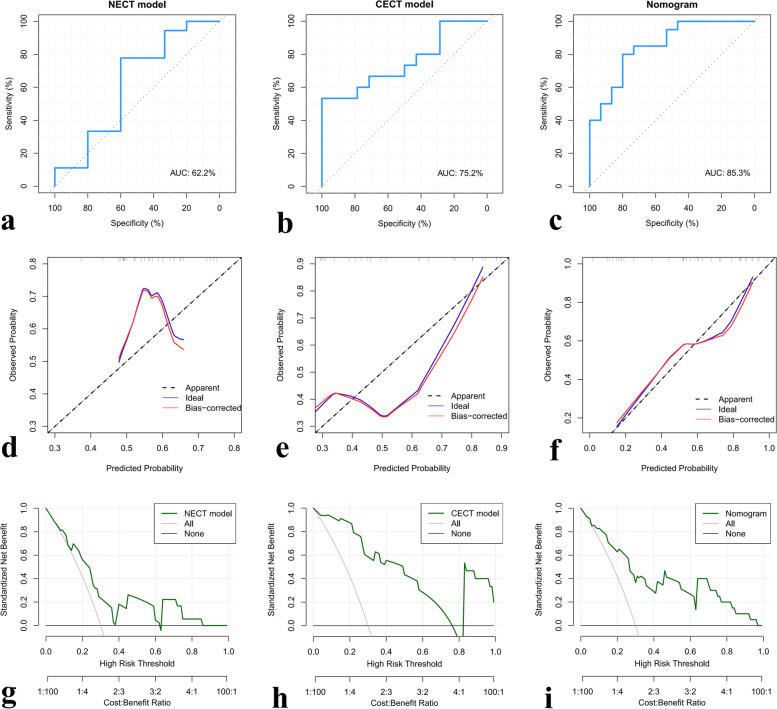
Receiver operating characteristic analysis of the NECT model, CECT model, and nomogram in predicting T790M resistance in the testing cohort (from **a** to **c**, respectively). Calibration curves showing the performance of the NECT model, CECT model, and nomogram in the prediction of T790M resistance in the testing cohort (from **d** to **f**, respectively). Decision curve analysis for predicting T790M resistance of NECT model, CECT model, and nomogram in testing cohort (from **g** to **i**, respectively). *CECT* Contrast-enhanced computed tomography, *NECT* Nonenhanced computed tomography

A radiomics nomogram was developed by integrating the rad-score of NECT and CECT in the training dataset (Fig. 4). The NECT, CECT, and nomogram were all significantly associated with the status of T790M (Fig. 5a–c). The ROC-AUCs of the NECT model were 0.730 (95% confidence interval [CI] 0.643–0.818) and 0.622 (95% CI 0.415–0.829) in the training and testing cohort, respectively. As for the CECT model, it yielded ROC-AUCs of 0.803 (95% CI 0.693–0.913) and 0.752 (95% CI 0.570–0.935) in the training and testing cohort. The predictive nomogram for the status of T790M based on the rad-score of NECT and CECT achieved an encouraging performance with a ROC-AUC of 0.828 (95% CI 0.717–0.937) in the training cohort, which was confirmed in the testing cohort with a ROC-AUC of 0.853 (95% CI 0.727–0.979). The cutoff values of NECT, CECT, and nomogram were 0.053, -0.472, and -0.087 in the training cohort. The accuracy, sensitivity, specificity, PPV, and NPV were also calculated in three models, respectively, and displayed in Table 2.

**Table 2:** Predictive performance of three models in the training and testing cohorts

Index	Training cohort			Testing cohort		
	NECT model	CECT model	Nomogram	NECT model	CECT model	Nomogram
ROC-AUC	0.730	0.803	0.828	0.622	0.752	0.853
Accuracy	0.698	0.797	0.812	0.697	0.759	0.800
Sensitivity	0.723	0.973	0.865	0.778	0.533	0.800
Specificity	0.672	0.556	0.741	0.600	1.000	0.800
PPV	0.701	0.750	0.821	0.700	1.000	0.842
NPV	0.695	0.938	0.800	0.692	0.667	0.750

*CECT* Contrast-enhanced computed tomography, *NECT* Nonenhanced computed tomography, *NPV* Negative predictive value, *PPV* Positive predictive value, *ROC-AUC* Area under the curve at receiver operating characteristic analysis

### Clinical utility

The NECT model, CECT model, and nomogram showed good agreement between the estimated probability of T790M positive and the observed rate of T790M positive in both datasets (Fig. 5d–f). In the results of Hosmer–Lemeshow statistic, there was no significant difference from an ideal fitting in NECT model (training cohort, *p* = 0.526; testing cohort, *p* = 0.551), CECT model (training cohort, *p* = 0.874; testing cohort, *p* = 0.730), and nomogram (training cohort, *p* = 0.230; testing cohort, *p* = 0.490). The nomogram showed a higher net benefit in differentiating T790M positive from T790M negative than the NECT and CECT model across the majority of the range of reasonable threshold probabilities by DCA (Fig. 5g–i).

### Follow-up and patient risk stratification

A total of 162 metastatic NSCLC patients were followed up to the data of May 1, 2023, successfully. At the end of the follow-up period, all patients developed T790M resistance. The resistant time was defined as the time between the date of receiving first-line TKIs (first- or second-generation EGFR TKIs) and the date of T790M being detected. The resistant time ranges from 1.4 to 105.7 months (mean 16.5 months, median 16.1 months).

**Fig. 6:**
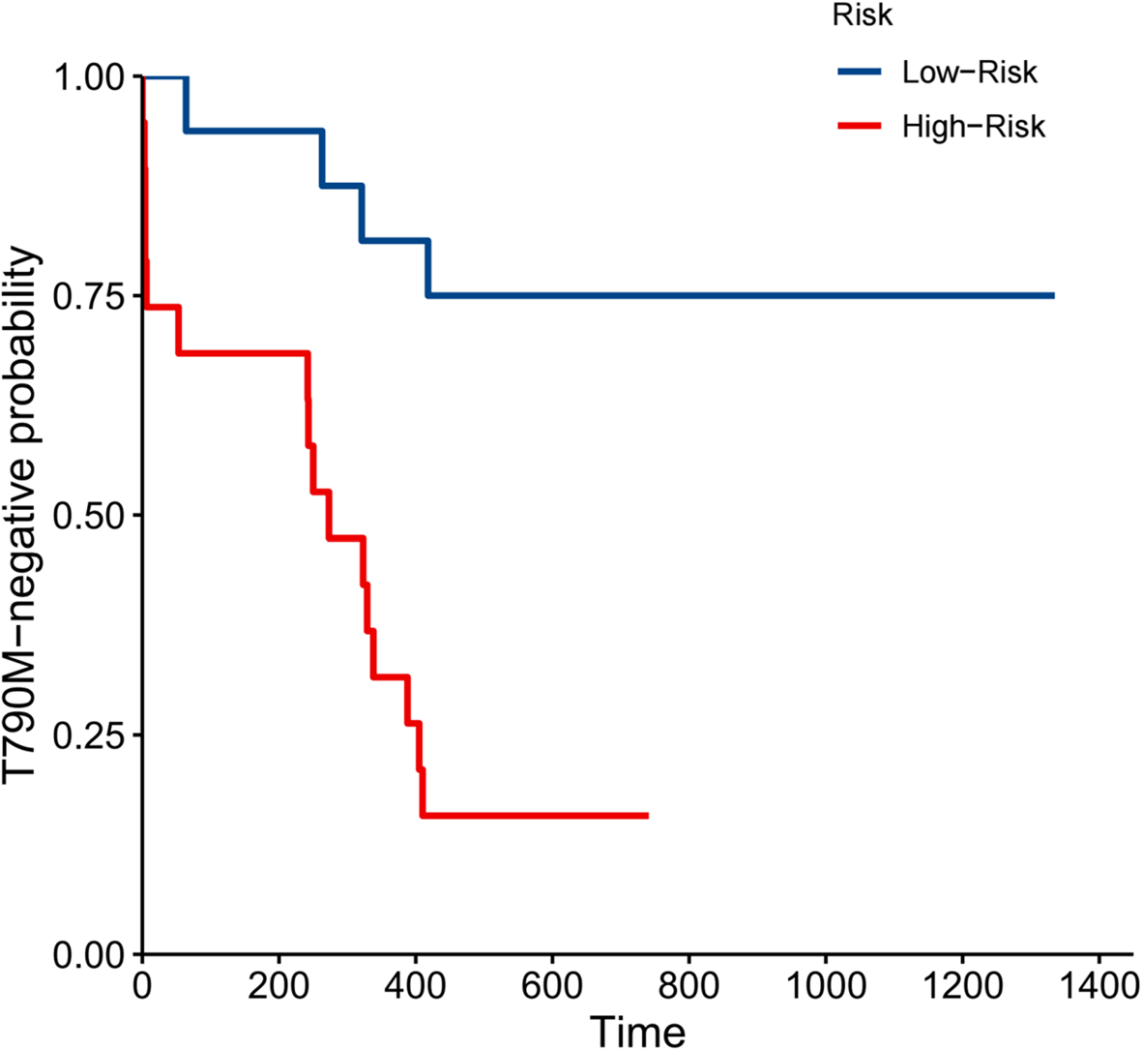
Kaplan–Meier survival curves of the time of T790M occurrence according to the risk score predicted by the nomogram in the testing cohort

The Kaplan–Meier curves of the proportion of patients with T790M negative were significantly different between the high- and low-risk groups (*p* = 0.00029, hazard ratio 6.210, 95% CI 2.037–18.930) (Fig. 6).

## Discussion

This study recruited and analyzed 162 metastatic NSCLC patients who were at initial diagnosis and received the first- or second-generation TKIs in the following treatment only. We firstly constructed radiomics models to identify patients who will develop T790M resistance within 14 months after TKIs and further explored a biomarker for risk stratification. Ultimately, this research demonstrated that the radiomics nomogram based on baseline NECT and CECT images could distinguish the patients developing T790M resistance in the early stage effectively.

Almost 51% oncogene-driven lung cancer patients are diagnosed with metastases at baseline, leading to a low 5-year survival rate of 21% [14]. Despite the early positive effect of first- or second-generation TKIs, the emergence of resistance is inevitable, and the time varies widely [4]. It was reported that resistance would emerge within 9 to 14 months after receiving TKIs [15]. Therefore, the fourteenth month was selected as the cut-off point to evaluate the resistant risk. In this research, we found that the nomogram depending on the rad-score of the NECT and CECT model achieved the best performance. And the rad-scores were remarkably higher in the high-risk group of T790M resistance.

The CECT model played a more important role than the NECT model in the nomogram. Besides, compared with the NECT model, the CECT model performed better with higher ROC-AUCs in the two cohorts. It revealed that the features extracted from CECT may be superior to that from NECT in predicting T790M-resistant risk which is in line with the previous study of Hong et al. [16]. They found that radiomics features extracted from CECT were better than that from NECT images in differentiating mutant and wild-type EGFR in advanced lung adenocarcinoma patients. It may be related to the reason that the tumor profile can be delineated well and the enhanced pattern of increased vascularity can be provided in the CECT images [16]. In contrast, Tang et al. [17] suggested that NECT features can distinguish T790M well when advanced NSCLC patients progressed. However, their research mainly focused on whether the lesion remained T790M mutation and did not discuss the time of emerging T790M. These conclusions indicated that NECT and CECT radiomics features perform differently in different conditions.

Our DCA curve showed that rather than assuming “all patients emerge T790M” or “all do not emerge T790M” across the majority of reasonable threshold probability, the nomogram model added more net benefit and had quality generalizability in clinical practice. In order to validate the risk-stratified ability of the nomogram, we extended the follow-up time. The nomogram model can successfully distinguish high-risk patients in emerging T790M mutation who need close monitoring and timely regulating targeted agents. And the Kaplan–Meier curves of the proportion of patients with T790M negative have a significant difference between the two groups with a hazard ratio of 6.210, which means the high-risk group is approximately six times more likely to develop T790M resistance than the low-risk group.

Some baseline clinical factors were analyzed in this study. For the total cohort, the initial EGFR mutation status has a significant difference between T790M-positive and -negative groups. It showed that patients who emerged T790M early had a higher proportion of EGFR 19Del mutation. This was in line with a previous study [17] showing that patients with 19Del mutation are more prone to developing T790M than those harboring L858R, whereas, as for the training and testing cohort, the difference was not significant which may be associated with the limited amount of cases.

Previous studies have demonstrated that radiomics has the potential to predict the mutation status of NSCLC [8, 13, 18–20]. Currently, a small part of studies have discussed the issue of T790M resistance mutation [12, 17, 21, 22]. Li et al. [12] have shown that radiomics based on multisequence MRI can differentiate NSCLC patients with brain metastases harboring T790M mutation from those who do not have T790M. Fan and colleagues [21] built a combined radiomics model using the features extracted from tumoral and peritumoral areas of brain metastasis to assess the T790M mutation status. Tang et al. [17] evaluated the value of chest CT radiomics in assessing T790M for advanced NSCLC patients after the failure of EGFR-TKIs, and they found that the NECT + CECT signature performed the best in assessing T790M. More and more studies have indicated that radiomics has the ability of risk stratification and prognostic prediction [23–25]. Zhao et al. [23] built a risk-stratified model to predict intracranial progression in ALK-mutant NSCLC patients with brain metastases after receiving ensartinib. The risk of emerging T790M mutation varies from different NSCLC patients after the first-line TKIs. Therefore, we hypothesized that the baseline CT radiomics features have the value in predicting T790M occurrence for metastatic NSCLC patients after TKIs.

There were still a few limitations in this study. Firstly, it is a retrospective research with a relatively small dataset. A prospective and multicentric study should be carried out in the future. Secondly, except for the T790M mutation, there are several other resistant mechanisms which were not analyzed in our research limited by the small number of cases. These infrequent resistance mutations will be evaluated in the continuous research. Thirdly, the optimal strategy for contrast enhancement in chest CT protocols is being debated [26]. Some guidelines and authors specifically recommend scanning after a delay of 20 − 35 s [27, 28]. The recruited patients in our study were scanned at a specific postinjection time delay. In future studies, we will use bolus tracking to decide when to start the scan.

In summary, this study showed that a nomogram based on baseline NECT and CECT images can effectively identify metastatic NSCLC patients who would emerge T790M mutation within 14 months after the first- or second-generation TKIs therapy. Besides, the nomogram also has the ability of risk stratification which is conducive to regulate targeted agents and develop individual follow-up strategies.

### Supplementary Information

**Additional file 1.** The formula of rad-score.

## Data Availability

The datasets used and/or analyzed during the current study are available from the corresponding author on reasonable request.

## Abbreviations

CECT: Contrast-enhanced CT
CI: Confidence interval
CT: Computed tomography
DCA: Decision curve analysis
EGFR: Epidermal growth factor receptor
NECT: Nonenhanced CT
NPV: Negative predictive value
NSCLC: Non-small cell lung cancer
PPV: Positive predictive value
Rad-score: Radiomics score
ROC-AUC: Area under the curve at receiver operating characteristic analysis
ROI: Region of interest
TKIs: Tyrosine kinase inhibitors
