# Supplementary material for: CT-based nomogram for early identification of T790M resistance in metastatic non-small cell lung cancer before first-line epidermal growth factor receptor-tyrosine kinase inhibitors therapy
Source: Eur Radiol Exp. 2023 Nov 2;7:64. doi: 10.1186/s41747-023-00380-7 (PMC10620367; doi:10.1186/s41747-023-00380-7)
Supplement: Supplementary file 1 — Additional file 1. The formula of rad-score. [file 41747_2023_380_MOESM1_ESM.docx]

**CT-based nomogram for early identification of T790M resistance in metastatic non-small cell lung cancer before first-line epidermal growth factor receptor-tyrosine kinase inhibitors therapy**

**ELECTRONIC SUPPLEMENTARY MATERIAL**

The formula of rad-score were listed as follows:

NECT: Rad-score =0.474497+ 0.088268 × Image-interpolated-Maximum - 0.222819×Wavelet-HLH-Glcm-Correlation + 0.011851 × Wavelet-LHL-First order-Maximum + 0.110121 × Wavelet-HHH-Glcm-MCC

CECT: Rad-score = 0.690362 + 0.051708 × Original-Glszm-GrayLevelVariance - 0.189200 × Log-sigma-2-0-mm-3D-Glszm-SizeZoneNonUniformityNormalized -0.137304 × Log-sigma-5-0-mm-3D-Glszm-SizeZoneNonUniformityNormalized + 0.207817 ×Log-sigma-5-0-mm-3D-Glszm-ZoneEntropy -0.05001× Wavelet-LLH-First order-Skewness + 0.067687 ×Wavelet-LHL-Gldm-LargeDependence LowGrayLevelEmphasis -0.06273 × Wavelet-HLL-First order-Skewness + 0.078150 × Wavelet-HHL-First order-Maximum + 0.321386 × Wavelet-HHL-First order-Skewness + 0.056522 × Wavelet-HHH-First order-Minimum
